# Septo-Optic Dysplasia Diagnosed in a Newborn Infant with Normoglycemia: The Importance of Thorough Physical Examination

**DOI:** 10.1155/2021/4836030

**Published:** 2021-11-13

**Authors:** Aishwarya Palorath, Ishita Kharode

**Affiliations:** ^1^Department of Pediatric Gastroenterology, University of Miami-Jackson Health System, 1601 NW 12th Ave, Miami, FL 33137, USA; ^2^Department of Pediatrics, Division of Pediatric Endocrinology, Richmond University Medical Center, 355 Bard Ave, Staten Island, New York, NY 10310, USA

## Abstract

A newborn male infant was admitted to the neonatal intensive care unit due to suspected sepsis. He was clinically stable with normal electrolyte levels on admission. However, he was noted to have micropenis and bilateral nonpalpable testes. Ultrasound imaging confirmed the presence of both gonads in the inguinal canal, with no Müllerian structures visualized. Laboratory examination revealed an undetectable random plasma cortisol level; subsequent ACTH stimulation testing confirmed adrenal insufficiency. Further testing revealed additional pituitary hormone deficiencies, and the infant was started on multiple hormone replacement therapies. Magnetic resonance imaging identified absent septum pellucidum, pointing of the frontal horns, and optic nerve hypoplasia. A diagnosis of septo-optic dysplasia was made based on this combination of findings. This case highlights the importance of thorough physical examination in newborn infants, which may reveal the only sign of underlying pathology in the absence of other concerning findings.

## 1. Introduction

Formerly known as de Morsier's syndrome, septo-optic dysplasia (SOD) occurs in 1 out of every 10,000 live births. It is a clinical diagnosis involving at least two of the following: midline brain defects (e.g., septum pellucidum and/or corpus callosum agenesis), optic nerve hypoplasia, and hypopituitarism. The phenotype is variable as most patients do not exhibit all three findings [1].

Hypopituitarism is the most common clinical finding in SOD, observed in 62–80% of patients [2]. Suggestive features include hypoglycemia, cleft lip and/or palate, micropenis, undescended testes, and prolonged jaundice [3].

We report a case of SOD diagnosed in a newborn infant who presented with micropenis and bilateral undescended testes. The infant was otherwise clinically well with persistently normal electrolytes, including normal blood glucose levels. However, his exam findings prompted an extensive diagnostic evaluation, and he was ultimately diagnosed with SOD based on laboratory and magnetic resonance imaging (MRI) results. This case report underscores the importance of a full physical examination in newborn infants, including a genital exam as atypical genitalia may be the only presenting sign of hypopituitarism and SOD.

## 2. Case Presentation

A newborn male infant was born at our institution at 41 weeks of gestation via normal spontaneous vaginal delivery to a 20-year-old G1P0 mother with negative prenatal serologies and normal prenatal ultrasounds. There was no known family history of endocrine conditions or midline defects or history of consanguinity between the parents. Birth weight was 3.71 kg. The patient was vigorous after birth, with Apgar scores of 9 at 1 minute and 9 at 5 minutes. However, due to maternal fever, the infant was admitted to the neonatal intensive care unit for suspected sepsis.

On physical exam, the infant was noted to have stretched penile length of 1.5 cm. Neither testis was palpable in the scrotum or inguinal canal. The remainder of the exam was unremarkable, with no cleft lip, cleft palate, or other dysmorphic features.

Initial laboratory evaluation showed normal electrolytes (sodium 137 mmol/L, potassium 3.5 mmol/L, and glucose 4.5 mmol/L). Scrotal ultrasound confirmed that both testes were present in the inguinal canal. No uterus or ovaries were seen. Pediatric endocrinology was consulted, and multiple laboratory tests were ordered to evaluate pituitary, adrenal, and gonadal functions. Pertinent results are presented in Table 1.

The patient's cortisol level resulted first as undetectably low, which prompted an ACTH stimulation test. A high-dose stimulation test was performed as it was unclear at that time whether cortisol deficiency was primary or secondary. Cortisol levels drawn at 30 and 60 minutes were both 24.8 nmol/L, consistent with suboptimal response and adrenal insufficiency. Within this context, the low insulin-like growth factor 1 and insulin-like growth factor-binding protein levels were suggestive of growth hormone deficiency. Growth hormone stimulation testing was not performed due to patient's young age. Thyroid function tests revealed central hypothyroidism, with inappropriately normal thyroid-stimulating hormone (TSH) level in the setting of borderline low free thyroxine (T4) level. There was no evidence of diabetes insipidus. The patient's sodium levels ranged 137–143 mmol/L, and mean urine output was 1.3 mL/kg/hour.

**Figure 1:**
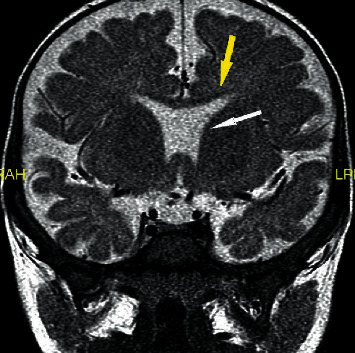
Absence of the septum pellucidum (white arrow) and pointing of frontal horns (yellow arrow) seen on MRI of the brain.

Given these findings, the patient was started on growth hormone, levothyroxine, and hydrocortisone therapy. Subsequent MRI revealed a small pituitary gland, absent septum pellucidum, and pointing of the frontal horns, consistent with a diagnosis of septo-optic dysplasia (Figure 1). Laboratory evaluation at three months of age, during minipuberty of infancy, again showed low total testosterone level (0.73 nmol/L).

Starting at six months of age, the patient underwent a short course of intramuscular testosterone therapy for micropenis, receiving 25 mg testosterone once monthly for a total of three doses. Stretched penile length after treatment completion was 3 cm, within 2.5 standard deviations for age. Our patient also underwent orchiopexy at 8 and 11 months of age, receiving perioperative hydrocortisone stress dosing for both surgeries.

Our patient has been evaluated by a pediatric ophthalmologist, who confirmed the presence of optic nerve hypoplasia. As of 18 months of age, he is tracking well with full extraocular muscle movements and has not, thus far, required eyeglasses. He has been receiving occupational and physical therapy since 9 months of age. The patient has been referred for genetic analysis but has not yet undergone evaluation.

## 3. Discussion

The presence of bilateral undescended testes in a genetically 46, XY male infant suggests either a defect in androgen action (such as partial 5-*α* reductase deficiency or partial androgen insensitivity syndrome) or a defect in androgen production, also known as hypogonadism [4].

Hypogonadism, in turn, can occur at either the central or peripheral level. Causes of congenital central, or hypogonadotropic, hypogonadism include pituitary defects such as Kallmann syndrome, septo-optic dysplasia, and adrenal hypoplasia congenita. The presence of midline defects such as cleft lip, cleft palate, solitary central incisor, and omphalocele makes a pituitary defect more likely [5]. Conversely, differential diagnoses for congenital hypergonadotropic hypogonadism include Klinefelter syndrome, partial gonadal dysgenesis, and enzymatic defects in adrenal testosterone biosynthesis [6].

Our patient presented with both undescended testes and micropenis, which is also associated with hypogonadism. Micropenis is defined as penile length less than 2.5 standard deviations below the mean. A stretched penile length measurement is vital in order to distinguish true micropenis from situations where the penile base is obscured [4].

Infants with suspected hypogonadotropic hypogonadism should undergo full pituitary hormone evaluation. Hypoglycemia, which is associated with both growth hormone and cortisol deficiencies, can be an additional suggestive finding. Interestingly, our patient was normoglycemic throughout his hospitalization, despite having multiple pituitary hormone deficiencies. The presence of low growth hormone and/or cortisol levels during hypoglycemia can assist in diagnosing deficiencies of these hormones [7, 8]. ACTH stimulation testing with cosyntropin is often used to confirm cortisol deficiency [3]. Thyroid function tests and serum/urine osmolality should also be monitored to assess for central hypothyroidism and diabetes insipidus, respectively [9, 10]. Importantly, pituitary hormone deficiencies can develop over time, so close endocrine follow-up is essential [1].

Hypogonadism itself can be confirmed during minipuberty of infancy, which occurs in boys up to age 6 months and in girls up to age 3 years [11]. Along with laboratory testing, MRI of the brain should be obtained to evaluate the morphology of the pituitary gland (including the presence of the posterior pituitary bright spot), corpus callosum, septum pellucidum, optic nerves, and optic chiasm [2].

Patients with hypopituitarism should receive replacement of any deficient hormones. Families of patients with adrenal insufficiency should be counseled on steroid stress dosing during times of illness and emergency [8]. Infants with micropenis can be treated with a short course of testosterone in order to increase penile length. A dosing regimen of 25 mg IM, given every 3 or 4 weeks for three doses, has been successfully implemented [2, 5].

Of note, testosterone therapy has been associated with decreased Sertoli cell function and spermatogenesis [12]. Recombinant FSH therapy during minipuberty, in combination with testosterone therapy, has been shown to improve Sertoli cell function in the short term, although longitudinal data are not available [13]. Topical dihydrotestosterone (DHT) has also been shown to increase penile length in patients with micropenis due to various genetic conditions, with minimal adverse effects. Again, data regarding precise dosing and long-term effects are not available [4, 14].

Patients with cryptorchidism whose testes have not descended by six months of age should undergo orchiopexy within the next year [15].

Patients with SOD should follow with a multidisciplinary team, with management aimed at treating pituitary hormone deficiencies, providing neurodevelopmental support, and monitoring for visual impairment [1].

## 4. Conclusion

A thorough physical examination is essential in newborn infants as it can reveal signs of a serious underlying condition. The presence of micropenis and bilateral undescended testes in a 46, XY infant should prompt assessment for hypogonadism. In cases of suspected hypogonadotropic hypogonadism, a full pituitary hormone workup should be undertaken along with brain imaging to further clarify the diagnosis. Septo-optic dysplasia is a rare but important cause of hypopituitarism, which necessitates management by a multidisciplinary team of specialists. Patients with SOD should be closely monitored for the potential development of additional pituitary hormone deficiencies over time.

## Figures and Tables

**Table 1:** Pertinent laboratory findings.

	Patient's age in days at the time of blood draw	Value	Units	Reference ranges for age
Plasma glucose	2	4.5	mmol/L	3.9–5.6
Random cortisol	4	<13.8	nmol/L	46.9–386
TSH	13	3.704	*µ*IU/mL	1.3–16
Free T4	13	11.6	pmol/L	10.8–63.9
IGF-1	5	<4.2	nmol/L	1.9–14.3
IGFBP-3	7	0.8	mg/L	1.11–3.18
LH	3	0.09	mIU/mL	0.02–7
FSH	3	0.0	mIU/mL	0.16–4.1
Total testosterone	3	2.1	nmol/L	2.6–3.5
Total testosterone (repeated level)	89	0.72	nmol/L	2.5–11.9
Karyotype	3	46, XY		

## References

[1] Webb E. A., Dattani M. T. (2010). Septo-optic dysplasia. *European Journal of Human Genetics*.

[2] Kurtoglu S. (2019). Neonatal hypopituitarism: approaches to diagnosis and treatment. *J Clin Res Pediatr Endocrinol*.

[3] Bosch i Ara L. (2021). Congenital hypopituitarism during the neonatal period: epidemiology, pathogenesis, therapeutic options, and outcome. *Front Pediatr*.

[4] Hatipoğlu N., Kurtoğlu S. (2013). Micropenis: etiology, diagnosis and treatment approaches. *Journal of clinical research in pediatric endocrinology*.

[5] Grumbach M. M. (2005). A window of opportunity: the diagnosis of gonadotropin deficiency in the male Infant1. *The Journal of Clinical Endocrinology & Metabolism*.

[6] Rodpraser W. (2020). Hypogonadism and cryptorchidism. *Frontiers in Endocrinology*.

[7] Grimberg A., DiVall S. A., Polychronakos C. (2016). Guidelines for growth hormone and insulin-like growth factor-I treatment in children and adolescents: growth hormone deficiency, idiopathic short stature, and primary insulin-like growth factor-I deficiency. *Hormone Research in Paediatrics*.

[8] Bowden S. A., Henry R. (2018). Pediatric adrenal insufficiency: diagnosis, management, and new therapies. *International Journal of Pediatrics*.

[9] Persani L. (2012). Central hypothyroidism: pathogenic, diagnostic, and therapeutic challenges. *The Journal of Clinical Endocrinology & Metabolism*.

[10] Abraham M. B. (2014). Efficacy of hydrochlorothiazide and low renal solute feed in neonatal central diabetes insipidus with transition to oral desmopressin in early infancy. *International Journal of Pediatric Endocrinology*.

[11] Boehm U., Bouloux P.-M., Dattani M. T. (2015). European Consensus Statement on congenital hypogonadotropic hypogonadism-pathogenesis, diagnosis and treatment. *Nature Reviews Endocrinology*.

[12] Liu P. Y., Baker H. W. G., Jayadev V., Zacharin M., Conway A. J., Handelsman D. J. (2009). Induction of spermatogenesis and fertility during gonadotropin treatment of gonadotropin-deficient infertile men: predictors of fertility outcome. *The Journal of Clinical Endocrinology & Metabolism*.

[13] Kohva E., Huopio H., Hietamäki J., Hero M., Miettinen P. J., Raivio T. (2019). Treatment of gonadotropin deficiency during the first year of life: long-term observation and outcome in five boys. *Human Reproduction*.

[14] Xu D., Lu L, Xi L (2017). Efficacy and safety of percutaneous administration of dihydrotestosterone in children of different genetic backgrounds with micropenis. *Journal of Pediatric Endocrinology & Metabolism: Journal of Pediatric Endocrinology & Metabolism*.

[15] Kolon T. F., Herndon C. D. A., Baker L. A. (2014). Evaluation and treatment of cryptorchidism: AUA guideline. *The Journal of Urology*.

